# Functional diversity of CTCFs is encoded in their binding motifs

**DOI:** 10.1186/s12864-015-1824-6

**Published:** 2015-08-28

**Authors:** Rongxin Fang, Chengqi Wang, Geir Skogerbo, Zhihua Zhang

**Affiliations:** CAS Key Laboratory of Genome Sciences and Information, Beijing Institute of Genomics, Chinese Academy of Sciences, Beijing, 100101 China; Bioinformatics Laboratory and National Laboratory of Biomacromolecules, Institute of Biophysics, Chinese Academy of Sciences, Beijing, China

**Keywords:** CTCF, Binding motif, DNA methylation, Chromatin interaction

## Abstract

**Background:**

The CCCTC-binding factor (CTCF) has diverse regulatory functions. However, the definitive characteristics of the CTCF binding motif required for its functional diversity still remains elusive.

**Results:**

Here, we describe a new motif discovery workflow by which we have identified three CTCF binding motif variations with highly divergent functionalities.

Supported by transcriptomic, epigenomic and chromatin-interactomic data, we show that the functional diversity of the CTCF binding motifs is strongly associated with their GC content, CpG dinucleotide coverage and relative DNA methylation level at the 12th position of the motifs. Further analysis suggested that the co-localization of cohesin, the key factor in cohesion of sister chromatids, is negatively correlated with the CpG coverage and the relative DNA methylation level at the 12th position. Finally, we present evidences for a hypothetical model in which chromatin interactions between promoters and distal regulatory regions are likely mediated by CTCFs binding to sequences with high CpG.

**Conclusion:**

These results demonstrate the existence of definitive CTCF binding motifs corresponding to CTCF’s diverse functions, and that the functional diversity of the motifs is strongly associated with genetic and epigenetic features at the 12th position of the motifs.

**Electronic supplementary material:**

The online version of this article (doi:10.1186/s12864-015-1824-6) contains supplementary material, which is available to authorized users.

## Background

The CCCTC-binding factor (CTCF) is an 11-zinc-finger protein that is functionally conserved in vertebrates, insects and nematodes [1]. It has been shown to be involved in various critical biological processes and has long been regarded as a versatile regulator. CTCF knockout mice exhibit early embryonic lethality prior to implantation [2, 3]. In adult organisms, the CTCF protein is ubiquitously expressed across most tissues, and its expression levels vary in a cell type-specific manner, suggesting a role in maintaining phenotypic diversity [4]. Furthermore, CTCF depletion results in dysregulated transcription of hundreds of genes in oocytes [5] and dramatically deregulates cell-cycle progression during T lymphocyte lineage commitment within the thymus [3]. Other studies have suggested that CTCF impacts cellular activity by playing diverse roles in gene regulation, including promoter activation/repression, genomic imprinting, enhancer blocking, and, most recently, long-range chromatin interactions [2, 4].

Recent genome-wide mapping of CTCF occupancy in multiple cell lines has enabled the identification of CTCF binding sequences [6–9]. Several hypotheses have been proposed to explain the uniquely versatile characteristics of CTCF based on the underlying binding motif sequence. The most widespread is the zinc-finger model, in which the capacity of CTCF to confer vastly different functions has been attributed to the interplay between the zinc-finger engagement and the underlying sequence [4]. Early studies in which zinc-fingers were depleted in a stepwise manner reported involvement of multiple zinc-fingers within a broad ~50 bp sequence [1], indicating that CTCF binding may be partially stabilized by interplay between peripheral zinc-fingers and other cofactors. Following this, a ~11–15 bp core consensus sequence was identified as bound by 4–5 central zinc-fingers [10]. A variation at the 12th bp position of the consensus sequence was found to be tightly associated with the DNA methylation level of the binding site which in turn was associated with remodeling of CTCF binding in immortalized cells [9]. Several studies have suggested that the functional diversity of CTCF is associated with its sequence variations [11, 12], but the mechanism by which sequence variation determines its function remains unexplored. Consequently, we sought to identify and characterize the variation in CTCF binding sequences and its relation to the CTCF functional spectrum. Using a newly developed motif discovery workflow, we were able to distinguish three different CTCF binding motifs. Integrative analyses of data on transcription factors, histone modifications, chromatin conformations and gene expression across multiple cell lines suggest distinct functionalities of these three CTCF binding sequence variations. In particular, our analysis revealed that CpG coverage and methylation status at the 12th position of the CTCF binding motifs have a marked effect on the colocalization of cohesin, which in turn implies that the variations in the CTCF binding sequence mediate different effects on chromatin interactions. To test this assumption, we examined the effects of the three CTCF binding sequence variations in relation to chromatin interactions, and found that chromatin interactions between promoters and distal regulatory elements tends to be mediated by CTCFs that bind to motifs with higher CpG content.

## Results and discussion

### CTCF binding sequence variations detected by a motif discovery workflow

It has been shown that the functional diversity of CTCF may be associated with the occupancy of the protein at its binding sites [11, 12]. This led us to the hypothesis that the functional diversity of CTCF may result from its binding affinity, and thus be influenced by the variations in the CTCF binding motifs. To obtain an extensive sample of the variations of CTCF binding motifs, we revisited the genome-wide data of chromatin immunoprecipitation of CTCF followed by high-throughput sequencing (ChIP-seq) from the ENCODE Project [13, 14]. While many of the CTCF binding sites were observed to be bound across all or most cell types, ~20–50 % of CTCF sites showed some level of cell type specific binding [6, 15]. However, cell type specific CTCF binding sites have recently been shown to be less occupied than constitutive sites [12], indicating that cell type specific binding of CTCF is less stable and weaker than constitutive binding, which implies the possibility that the detection of cell type specific CTCF binding events may have a higher false positive rate. In order to obtain high confidence CTCF binding sites, we extracted CTCF ChIP-seq peaks that appeared in all the 12 tested cell lines [16–18] (Additional file 1: Table S1) and regarded these as “constitutive” binding sites. A total of 12,761 constitutive CTCF binding sites were detected by MACS [19] with a threshold FDR < 0.01. Sequences of 200 bp in length centered at the summit of each CTCF binding peak were extracted and pooled for motif discovery. In the rest of this paper, if not stated otherwise, the analysis was performed on the aforementioned constitutive CTCF binding sites.

**Fig. 1:**
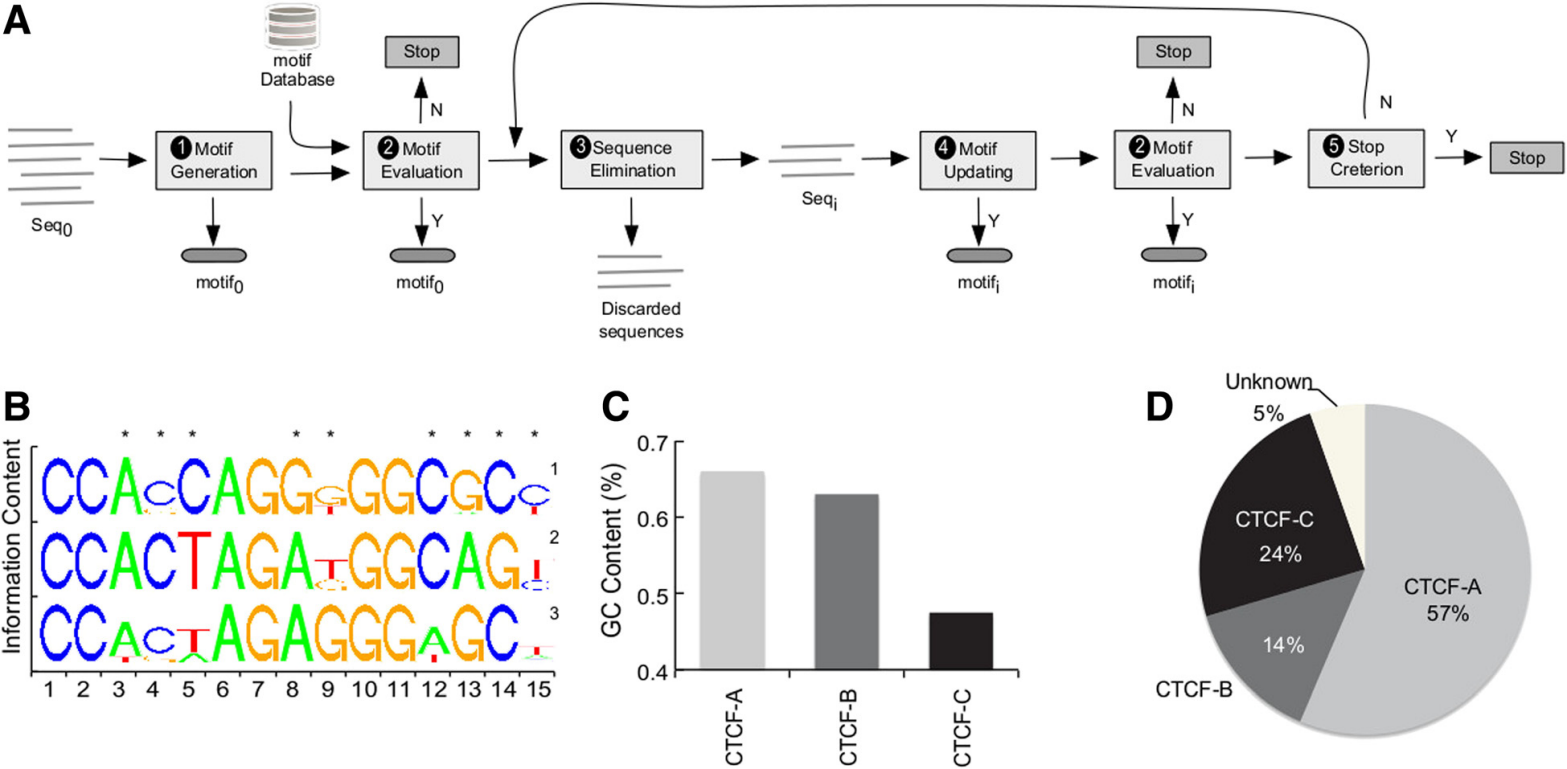
Motif Discovery Workflow and three detected CTCF sequence variations. **a** Cartoon of the motif discovery workflow (see Methods for details and Table 1 for the pseudocode of the algorithm). **b** Motif Logo for three core CTCF consensus sequences (1, 2, 3 for CTCF-A, CTCF-B and CTCF-C motif, respectively). The stars (*) in the first row indicate the most informative sites. **c** Average GC content at the most informative sites of the three CTCF binding sequence variations. **d** Distribution of three CTCF binding sequence variations

Different from previous motif generation methods that attempt to obtain a maximal description for all pooled sequences as a whole [20, 21], our workflow searches for multiple motifs, balancing the number of sequences each motif represents and the divergent nature of the recognition motifs of a given binding protein. In other words, our workflow detects motifs that represent the consensus of mutually exclusive subsets of pooled sequences, and the motifs consequently represent sequence subsets that could be very different. Our workflow consists of five major steps: (1) motif generation; (2) motif evaluation; (3) sequence elimination; (4) motif updating; and (5) a stopping criterion (Fig. 1a, Table 1 and Additional file 2).

**Table 1:**
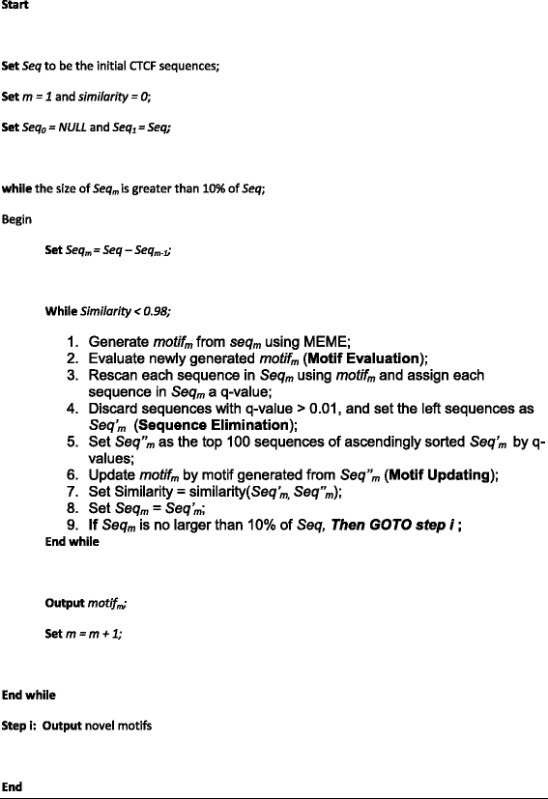
Pseudo-code for the motif discovery workflow. The details for Motif Evaluation, Sequence Elimination and Motif Updating can be found in the Additional file 2

We applied this workflow to the CTCF binding sequences described above, and identified three distinct CTCF core motifs of high confidence (Fig. 1b, *E*-values < 2.0E-457, < 3.0E-609 and < 2.1E-519:; *q*-values < 7.0E-10, < 1.0E-4 and < 7.0E-4, for the three motifs, respectively). The details concerning iteration and convergence of the process are shown in Additional file 3: Figure S1. We named the three CTCF binding sequence variations as CTCF-A, CTCF-B and CTCF-C, respectively (Additional file 4: Dataset S1). CTCF-A motifs constituted the largest fraction of the motifs (57 %), while 24 and 14 % of the CTCF binding sites contained the CTCF-B and CTCF-C motifs, respectively (Fig. 1d). The remaining 5 % of the binding sites did not fall into any of the three categories above, and were excluded from subsequent analyses. At the DNA sequence level, the three motifs did not show substantial divergence (Fig. 1), although the GC content in the informative sites of CTCF-A was moderately larger than in the CTCF-B and CTCF-C motifs. We then extracted the flanking region [−100 bp, +100 bp] centered at each CTCF binding motif, and calculated the binding intensity by counting the number of ChIP-seq reads that mapped within the flanking region. The binding affinity differed significantly among the three motifs (Additional file 5: Figure S2), suggesting three CTCF motifs may confer distinct functionalities. We therefore sought to investigate whether there is functional divergence among CTCF binding sites containing the three different motifs.

### Actively expressed genes are predominantly associated with CTCF-A

**Fig. 2:**
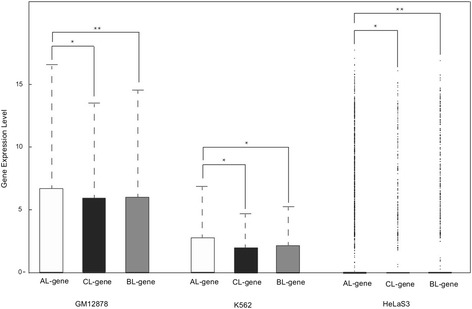
Expression of CTCF-linked genes. The distribution of associated gene types for the different CTCF binding sites is shown for all three cell types. Active genes are more likely to be linked with CTCF-A, compared with CTCF-B and CTCF-C binding sites. (“*” and “**” indicate *P*-value < 0.01and *P*-value < 0.001, respectively). The expression levels are showed in logarithmic transformation

**Fig. 3:**
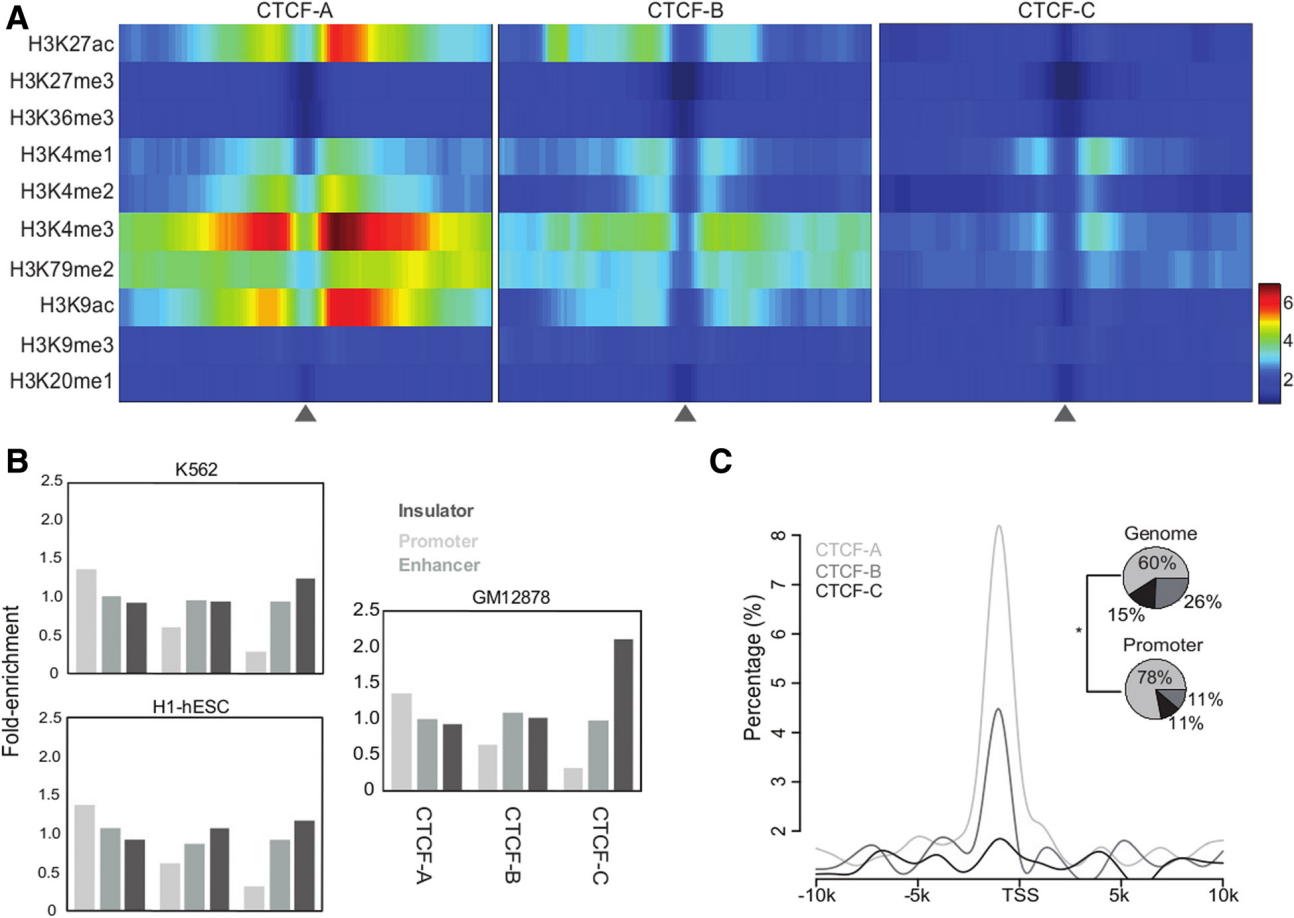
Genome-wide distribution of three CTCF binding sequence variations. **a** The heatmap shows fold-enrichment of 10 histone modifications in the flanking regions ([−1.5kbp, +1.5kbp]) of the three CTCF binding site variations (see Methods) (“▲” indicates the center position of CTCF binding sites). **b** Distribution of three CTCF binding sequence variations in promoters, enhancers and insulators as annotated by the ENCODE Project. **c** The distributions of three CTCF binding sequence variations in TSS flanking regions ([−10kbp, +10kbp]). The pie charts show the distributions of the three CTCF binding sequence variations in the whole genome and in promoter regions ([−2500 bp, +500 bp] to TSS); the star (*) indicates a significant difference between the two by the hypergeometric test (*p*-value < 1E-50; see Additional file 8: Figure S4 for distribution of three CTCF binding sequence variations within other regions)

To investigate the association between gene expression activity and the occupancy of CTCF binding sites, we used a set of histone modification data from the Broad Institute [22] available from the ENCODE project (Additional file 6: Table S2), in combination with counts of histone marks in the flanking regions of each CTCF binding site (Fig. 2, Fig. 3a). We observed that regions close to CTCF-A binding sites were highly enriched for chromatin features that have been associated with active regulatory genome elements. For example, an enrichment of H3K4me3 and H3K9ac is considered a strong indication of an active promoter [23, 24], and an enrichment of H3K27ac separates active from poised enhancers [25]. In contrast, the vicinity of CTCF-A sites were depleted of repressive chromatin marks, such as H3K27me3 [26]. We did not observed any significant enrichment of particular chromatin features in the vicinity of CTCF-B and CTCF-C binding sites. This pattern was even more pronounced for tissue specific CTCF binding sites (Additional file 7: Figure S3), in that tissue specific CTCF-A binding sites showed a higher frequency of active chromatin marks than constitutive CTCF-A binding sites (Wilcoxon-Rank-Sum test *p*-value = 0.037 and 0.012 for H3K27ac and H3K4me3).

These results prompted us to investigate the association between gene expression and the occupancy of CTCF binding sequence variations. We divided all the coding genes into four groups based on CTCF occupancy in the flanking regions of their transcriptional start sites (TSS), referred to as CTCF-A-Linked (AL), CTCF-B-Linked (BL), CTCF-C-Linked (CL) or ‘other’ genes. Excluding ChIP-seq peaks that did not correspond to any of these three CTCF binding motifs, we assigned each of the 9822 genes annotated by ENSEMBLE into a group corresponding to its nearest CTCF binding motif within a [−50kbp,+50kbp] region of its TSS, or, if none, as ‘other genes’. We examined the transcriptional activities of the genes of each group in the GM12878, K562 and HeLaS3 cell lines based on RNA-seq data available from ENCODE [27]. Most of the examined genes were transcriptionally silent; however, in all three cell lines, we found that the expression levels of AL genes were, in general, higher than those of BL (Wilcoxon test, *p*-value = 1.93E-4, 1.66E-2 and 4.96E-4 for GM12878, K562 and HeLaS3 cells, respectively) and CL genes (Wilcoxon test, *p*-value = 2.12E-3, 1.65E-2 and 1.45E-2 for GM12878, K562 and HeLaS3 cells, respectively). Taken together, these results suggest that CTCF-A binding sites are more frequently involved in active gene transcriptional regulation than the two other types of sites.

### Functional diversity of CTCF sequence variations

As the next step, we studied the overlap between the CTCF sites and annotations of regulatory regions. Twelve percent of all CTCF binding sites were located within 3 kb regions ranging from 2500 bp upstream to 500 bp downstream of annotated TSSs of coding genes (defined as promoter regions) while the remaining 88 % of the sites were evenly distributed (44 and 44 %) between intergenic and intragenic locations (Additional file 8: Figure S4), which is consistent with a previous mapping [28]. We further considered the overlap with regulatory regions annotated by the ENCODE chromatin state pattern [29, 30]. Specifically, we studied the overlap of CTCF binding sites with 6 types of genomic annotations, including active promoters, weak promoters, poised promoters, strong enhancers, poised enhancers, and insulators [29]. To further refine the annotation of the chromatin states, we pinpointed promoter, enhancer and insulator elements by adding additional restrictions of location or cofactors. For example, a DNA segment with a promoter-like chromatin state is regarded as a true promoter only if it also locates within [−2000 bp, +500 bp] of an annotated TSS. Likewise, enhancers and insulators needed to be colocalized with p300 [31] and cohesin [32, 33], respectively, the data on p300 and cohesin binding sites were obtained from the ENCODE ChIP-seq dataset [17]. In all three cell lines examined, the three CTCF motifs exhibited pronounced differences in inferred functionality. Particularly, CTCF-A binding sites exhibited a strong tendency to overlap with promoters (hypergeometric test *p*-value < 2E-53, < 9E-57 and < 2E-55 for GM12878, K562 and H1-hESC, respectively), while CTCF-C binding showed enrichment for overlap with insulators (hypergeometric test *p*-value < 7E-28, < 2E-19 and < 1E-32 for GM12878, K562 and H1-hESC, respectively). On the other hand, CTCF-B did not show enrichment for any of the chromatin states we tested (Fig. 3b), and we have for this reason, mainly compared CTCF-A and CTCF-C in the rest of the paper.

**Fig. 4:**
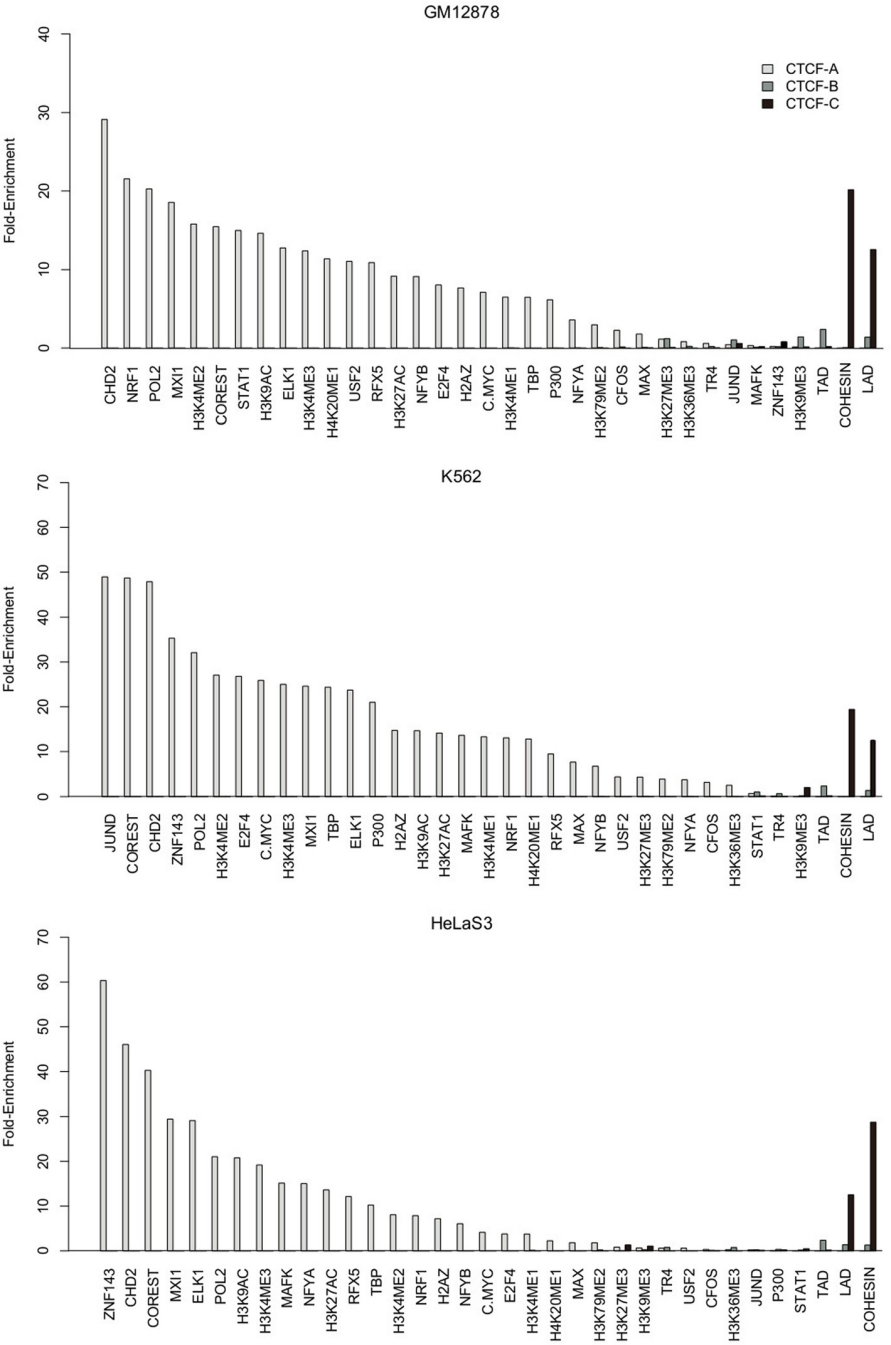
Fold-enrichment of functional genome elements within binding regions of the three CTCF variations in **a** GM12878, **b** K562 and **c** HeLaS3, cell lines

We can make several predictions based on the inferred functionality distributions. First, considering the enrichment of CTCF-A binding sites in promoter regions (Fig. 3c) and the strong association with gene expression activity (Fig. 2), we would expect a higher incidence of colocalization between CTCF and transcription factor (TF) binding. To test this, we integrated ChIP-seq data for 20 different transcription factors available from ENCODE (Additional file 9: Table S3) [13, 14] and calculated the fold-enrichment of colocalization of these transcription factors with the CTCF binding sites compared to the input signal (see Methods). As shown in Fig. 4, in all three cell lines we tested, we observed that binding of CTCF to CTCF-A sites colocalized with binding of most of the TFs we tested in all three cell lines. Second, the engagement of CTCFs in mediating transcriptional insulation has been found to be coupled with cohesin [33]; therefore, we can expect a subset of CTCF binding sites to be colocalized with cohesin. In fact, the results show a strong association between CTCF-C bindings and cohesins (binding of cohesin was defined as the overlapping peaks of its two subunits, Rad21 and SMC3) (Fig. 4). We also observed a strong association between CTCF-C bindings and Lamina associated domains (LADs), which also represent a repressive chromatin environment [34].

In summary, CTCF-A binding regions were enriched for active histone modifications and tended to appear within promoter regions. As such, they presumably consist of active regulatory elements in enhancers and promoters. In the absence of active histone marks and enrichment for insulator-like genome segments, CTCF-Cs binding regions possibly function as enhancer-blocking insulators.

### The sequence and DNA methylation variation at the 12^th^ position of the CTCF binding motif

We next asked what characteristics of the motifs might influence the divergence of their functionality. Given the differences in GC content among the three motifs, it is possible that differential DNA methylation levels at the CpG dinucleotide sites may result in different CTCF binding affinities. Two positions in the CTCF recognition motif have been reported to show an enrichment of CpG dinucleotides, with a strong association between DNA methylation level and CTCF occupancy [9]. To investigate the association between DNA methylation levels at the two sites and the potential functions of the three sequence variations, we extracted the CpG methylation status in [−50 bp, +50 bp] regions centered at each CTCF binding motif [35].

**Fig. 5:**
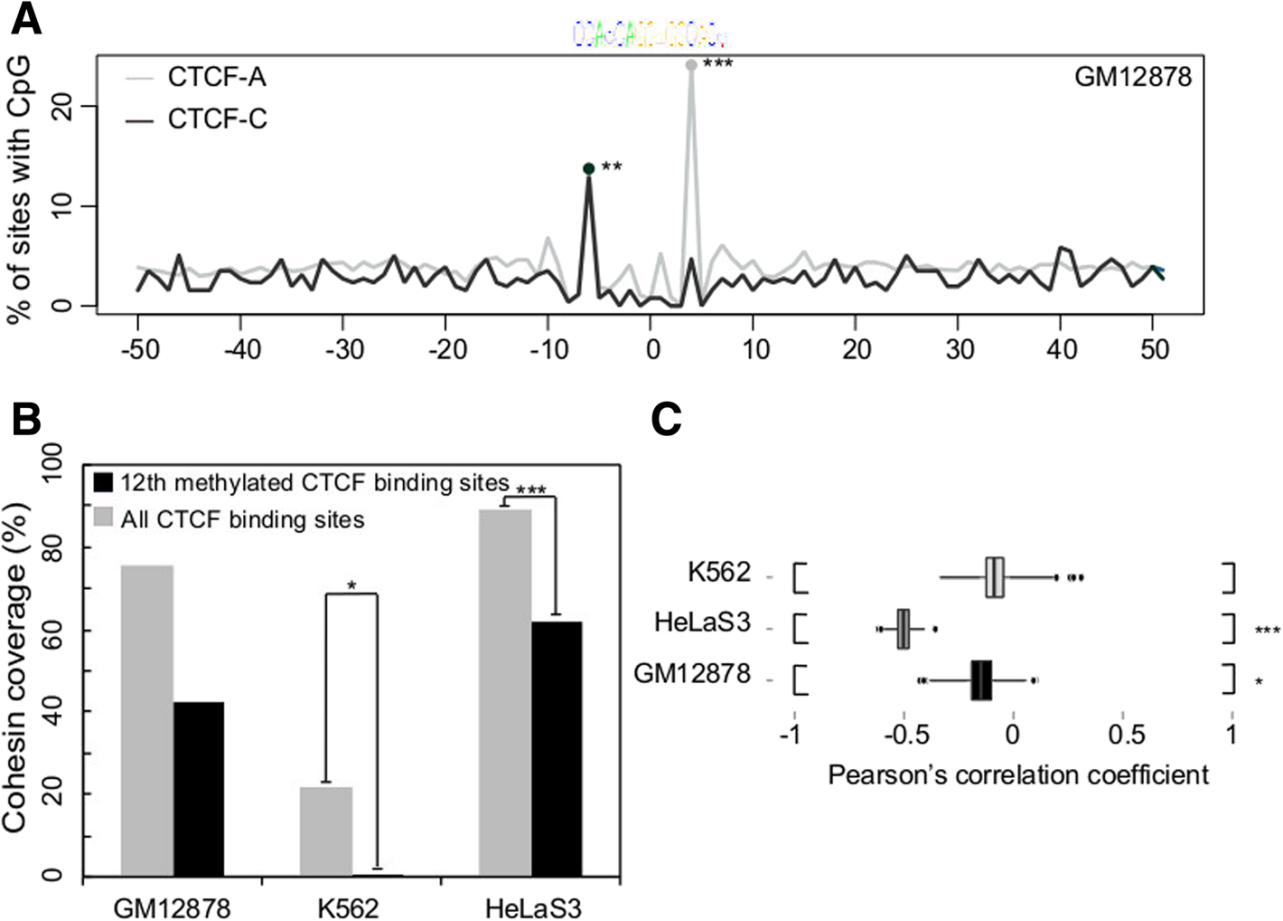
CpG coverage and methylation status at the 12th position of the CTCF motifs. **a** CpG coverage (%) distribution within regions [−50 bp, +50 bp] of the center of CTCF-A and CTCF-C binding sites in GM12878 (see Additional file 10: Figure S5 for K562 and HeLaS3). See Additional file 11: Figure S6 for the corresponding DNA methylation distribution. **b** Depletion of cohesin (represented by overlapping binding peaks of SMC3 and Rad21) at CTCF binding sites. The black and gray bars represent cohesin coverage at all CTCF binding regions and at CTCF binding sites that are methylated (methylation level >20 %) at the 12th position, respectively. **c** Correlation (Pearson’s correlation coefficients) between the ChIP-seq read counts for cohesin (Rad21) and the DNA methylation level at the 12th position of the CTCF binding sites across the three cell lines. The stars (*) indicate the statistical significance in each test (***, *p*-value < 1E-5, **, *p*-value < 1E-2, * *p*-value < 5E-2)

CTCF-A sequence regions have a relatively high overall CpG content and high DNA methylation levels at the 12^th^ position. Since the overall GC content varies among the CTCF-A, −B and -C sequences, it was not surprising to find that of all CpG dinucleotides detected in the three examined cell lines (13,714, 18,105 and 15,241 in GM12878, K562 and HeLaS3, respectively), CTCF-A sequences had much higher overall CpG levels than CTCF- B and -C sequences (Wilcoxon test *p*-value < 5e-12, < 5e-13 and < 5e-10 for GM12878, K562 and HeLaS3, respectively). In agreement with Wang et al. [9], we also observed ultrahigh enrichment of CpG dinucleotides at the 12th position of CTCF recognition sequences particularly in the CTCF-A subgroup (5-fold over that in CTCF-C, *p*-value < 1e-14; Fig. 5a and Additional file 10: Figure S5). Given the high CpG level at the 12th position of the CTCF-A sequences, we would also expect a correspondingly high DNA methylation level. Indeed, the DNA methylation level at the 12th position of CTCF-A binding sites was relatively high compared to other CpG sites (Additional file 11: Figure S6).

One puzzling observation is that both GC content and DNA methylation levels appear relatively high in and around CTCF-A binding sites located in transcriptionally active regions. However, this may in part be explained by the fact that the overall DNA methylation level at the constitutive CTCF binding sites is quite low compared to inactive or unbound CTCF sequences. When comparing the DNA methylation levels of CTCF-A binding sites with control regions, which were defined as the genome segments with high confident CTCF-A motifs but with little CTCF binding signal (i.e., a CTCF signal is less than bottom 5 % across all CTCF CHIP-seq peaks; Additional file 12: Figure S7), we found that the DNA methylation levels at CTCF-A binding sites were much lower than in the control regions.

If the regulation of functional diversity among CTCF binding sequence variations is influenced by the relative DNA methylation level at the 12th position, we would expect different associations between the DNA methylation levels at this position and key cofactors. To test this hypothesis, we divided the CTCF binding sites into two groups labeled as DNA “methylated” and “unmethylated” according to the DNA methylation level at their 12th position. Thus, if the DNA methylation level at the 12th position of a CTCF binding site was found to be greater than 20 % [36], this binding site would be classified as “methylated” otherwise the binding site is classified as “unmethylated”. As previously reported [9], we observed that the binding intensity of CTCF is negatively correlated with the methylation level at the 12th position of the binding sites (Additional file 13: Figure S8). We also examined the colocalization of cohesin with methylated and unmethylated CTCFs. Cohesin is one of the most important cofactors of CTCF, and exhibits distinct functionalities in the presence or absence of CTCF [32]. Because the peak numbers and genome wide distributions of ChIP-seq reads from the two subunits of cohesin (Rad21 and SMC3) are substantially different, we took only the overlapping binding regions of Rad21 and SMC3 to be representative of cohesin binding sites, investigating whether the colocalization of cohesin with CTCF was associated with DNA methylation level at the 12th position, we found that cohesin was highly depleted from methylated CTCF binding sites (Fig. 5b), the finding that is consistent across the three cell lines we examined (hypergeometric test *p*-value < 0.001, < 8E-7 and < 3E-13 for GM12878, K562 and HeLaS3, respectively). We also tested the effect of methylation at other sites within the CTCF core binding region [−10 bp, +10 bp], however, the 12th position was the only site that showed statistically significant depletion of cohesin occupancy in all three cell lines. Moreover, estimates of the cohesin binding affinity (as inferred from ChIP-seq read counts of Rad21 from representative cohesin binding sites) in GM12878 and HeLaS3 cell lines were negatively correlated with the DNA methylation level at the 12th position of the associated CTCF binding sequences (bootstrap test, *p*-value < 5E-2 and <1E-5 for GM12878 and HeLas3, respectively; see Fig. 5c and Methods). The data thus indicate that the co-binding of cohesin with CTCF is, to some degree, negatively related to DNA methylation at the 12th position of the CTCF binding site.

Renda and colleagues demonstrated that high-affinity binding to a 12 bp variation of the CFCT consensus sequence involved only 4–5 specific zinc-fingers of the CTCF [10]. Ren and colleagues reported that 17 % of all evolutionary nucleotide changes in the CTCF binding sites took place as C-to-T substitutions as a unique nucleotide change at the 12th position [6]. Our results, when combined with those of others [10, 6], imply that the methylation level at the 12th position of CTCF binding sequence may alter the binding environment, resulting in different zinc-finger binding and, in turn, recruiting different cofactors that ultimately leads to divergent functionalities.

### Functional diversity of CTCF binding sequence motifs is reflected in the genome 3D structure

**Fig. 6:**
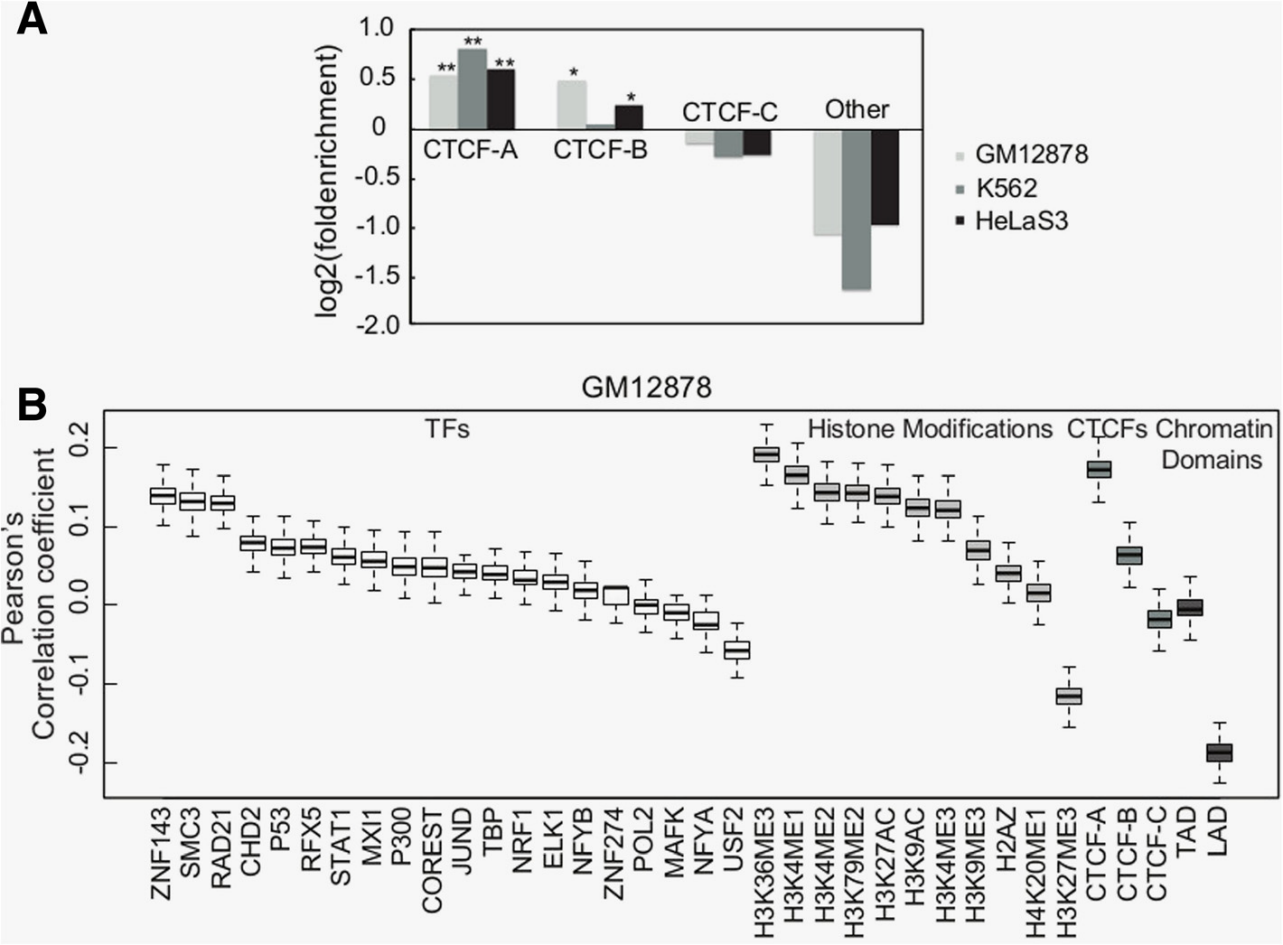
Association between the three CTCF binding sequence variations and functional elements. **a** Enrichment of TSS-distal chromatin interactions in the three CTCF binding sequence variations. The symbols, “*” and “**” indicate the significance of hypergeometric tests for *p*-value < 1E-2 and *p*-value < 1E-10, respectively. **b** Distribution Correlations between TSS-distal chromatin interactions and 36 genomic features in GM12878 cells. (see Additional file 14: Figure S9 for K562 and HeLaS3). The panel shows the distribution of Pearson’s correlation coefficients

Because the CTCF protein is important for mediating chromatin-chromatin interactions [4, 37], we next asked what connection might exist between CTCF binding site variation and the DNA loops in which they are involved. To address this question, we revisited the chromatin interactions database in ENCODE, which is based on data obtained with the Chromosome Conformation Capture Carbon Copy (5C) method [37, 38]. These data contain chromatin interactions between 628 TSS-containing fragments and 4443 distal restriction fragments covering the ENCODE pilot project regions representing 1 % (30 Mb) of the genome for the three cell lines examined above (GM12878, K562 and HeLaS3). We first used the sequence scanning tool FIMO with default settings to search each distal fragment for CTCF-A, −B and -C motifs [39]. Each fragment with the CTCF binding type was tagged by which had the highest confidence for this fragment (see Methods). In all three cell lines, we observed that fragments tagged with CTCF-A were enriched for TSS-distal chromatin interactions, while CTCF-C-linked fragments showed depletion of TSS-distal interactions compare to CTCF-A-linked fragments (Fig. 6a). CTCF-B, however, was enriched for TSS-distal interactions in HelaS3 and GM12878, but not in K562. In agreement with recent works showing that CTCF is involved in mediating long-range interactions [2, 4, 40], distal fragments lacking CTCF occupation also showed a low tendency to form loops. We further examined the correlation of 11 histone modifications (Additional file 6: Table S2), 20 DNA-binding factors (Additional file 9: Table S3) from ENCODE and 2 chromatin domains (LAD: Lamina Associated Domains and TAD: Topological Associated Domains Boundary) with loop formation (see Methods), and observed that TSS-distal looping is more strongly correlated with CTCF-A binding than with most of these other biological elements (Fig. 6b, see Additional file 14: Figure S9 for K562 and HeLaS3).

CTCF-A binding sites appeared more involved in the mediation of DNA looping between TSSs and enhancer-containing distal fragments than did CTCF-C binding sites. We found that cohesin (Rad21 and Smc3) was consistently and highly positively correlated with TSS-distal looping in all three cell lines. Although cohesin was previously thought to act as insulator when colocalized with CTCF [32, 33], our results are in agreement with recent genome wide studies showing an import role for cohesin in establishing enhancer-promoter interactions [33, 41]. We also observed enrichment of the NFYA and NFYB factors (Fig. 4) around the CTCF-A binding regions. These two proteins have been shown to interact with the CCAAT/Enhancer Binding Protein (C/EBP) which regulates gene expression [42]; therefore we further examined the enrichment of regulatory genome elements in the distal fragments. Compared to all fragments, the distal fragments mainly occupied by CTCF-A sequences exhibited higher enrichment for enhancer-related marks (*P*-value < 0.002, 0.05 and 0.004 for P300, H3K4me1 and annotated enhancers ([29, 30], respectively; Additional file 15: Figure S10), but not for H3K4me3.

## Conclusions

To determine divergent DNA recognition motifs of CTCF in various functional genomics contexts, we developed a novel motif discovery workflow focusing on the balance between the number of sequences a motif represents and the internal divergence within the sequence subgroups. By applying the workflow to the ENCODE ChIP-seq data set, we identified three CTCF core motif variations. The expression activity patterns of the genes flanking the three CTCF motifs showed significant correlations with the GC content and the CpG enrichment of the motifs. We also detected a strong association among the presence of the CTCF-A motif and enhancers and promoters defined by histone marks, and we further demonstrated that this association was supported by chromatin structural data from chromatin conformation capture based experiments. The functional divergence of the motifs was further associated with possible genetic or epigenetic variations, in particular with the CpG dinucleotide coverage at the 12^th^ position of the core binding motifs and the relative DNA methylation level at this site, and the latter two features were also strongly and negatively correlated with the colocalization of CTCF with cohesin, the key cofactor of CTCF when it functions as an insulator protein.

These results suggest that the variation in DNA methylation level at a single CpG site of the CTCF’s recognition motif has a determining influence on the divergence of its functions. Alternative preferences for critical cofactors, e.g. cohesin, among the different motifs suggest potentially multiple molecular mechanisms for the CTCF functionalities. The workflow we have introduced provides a new analytical tool for the studies of multifunctional DNA binding proteins, particularly for those whose functional classification is not yet clearly defined.

## Methods

### Minimal motif discovery workflow

Briefly, the workflow iteratively optimizes an object with the best motif score that can be found in a sub-dataset. See Fig. 1a, Table 1 and supplemental text for details.

### Dataset

Most of the data used were from the ENCODE Project [13, 14]. CTCF occupancy was derived from ChIP-seq data from two independent sources [16, 17]. The ChIP-seq histone modification signals across the three cell lines (GM12878, K562 and HeLaS3) were generated by Broad/MGW ENCODE group [22, 29]. The chromatin state segmentation for each of the three cell lines was acquired by computationally integrating ChIP-seq data for nine factors plus input using a Hidden Markov Model [29, 30]. The genome-wide binding sites for 24 different TFs were determined by ChIP-seq [17]. The DNA methylation profile was generated by Meissner et al. and assayed at more than 500,000 CpG dinucleotides in the genome, using Reduced Representation Bisulfite Sequencing (RRBS) as a part of the ENCODE Project [35]. The chromatin interaction data were generated using the Chromatin Conformation Capture Carbon Copy (5C) method from the Dekker Lab [37, 38].

### Histone modification fold-enrichment

To obtain the most reliable CTCF binding sites, we determined the binding sites by a combination of CTCF ChIP-seq data and motif scanning. Briefly, the peaks of CTCF ChIP-seq data were first called by MACS [19] with threshold FDR < 0.01. Next, the gained peak regions were scanned by the three CTCF motif variations generated by our workflow (Fig. 1a, Table 1) using motif scan software FIMO [39]. Then, a CTCF binding site in the peak region was defined as the motif instance locus having a FIMO E-value < 0.01. Regions [−1500 bp, +1500 bp] centered at each CTCF binding motif were extracted and partitioned into 150 bins of 20 bp each. The signal strength of each bin was retrieved from the original ENCODE bigwig files (Additional file 6: Table S2). The fold-enrichment of a histone modification at each bin was defined as:

$$ {S}_{ij}/{C}_j $$

Where *S*_*ij*_ is the strength of the *i-*th histone modification within the *j-*th bin, and *C*_*j*_ is the strength of Input in the *j-*th bin.

### CTCF colocalization fold-enrichment

The fold-enrichment of 36 biological elements (Additional file 6: Table S2 and Additional file 9: Table S3) for colocalization with CTCF binding sites is defined as follows:

$$ {P}_i/{C}_i $$

where *P*_*i*_ is the percentage (%) of CTCF binding sites overlapped by biological element *i*. and *C*_*i*_ is the percentage of control regions overlapped by the same biological element *i* as *P*_*i*_. Control regions were the peaks of the ChIP-seq Input experiment also called by MACS with FDR < 0.01; the CTCF overlapped regions were discarded. Features were determined to be colocalized with CTCF binding sites if they were overlapped by at least one nucleotide.

### Pearson correlations between genomic elements and looping

Detected looping events are very sparse in the 5C data; only 1.2 % of all distal-TSS pairs contain a significant loop (positive set [37]). Therefore, to correlate looping events with genomic elements, it is necessary to take the sparseness, i.e., the huge number of distal-TSS pairs with no significant 5C loop (negative set) into consideration. We used a bagging strategy to down-sample the negative observations to estimate the distribution of Pearson’s correlation coefficient (PCC) between genomic elements and 5C looping. In detail, we randomly sampled the same number of distal-TSS pairs with no 5C loops to form a control dataset, and 1000 such control datasets were generated, and the PCC distribution for each genomic element was calculated for the 1000 combined subsets.

### Availability of supporting data

All our data have been made available as the online supporting materials.

### Supporting information

Detailed information on the minimal motif discovery workflow.

## Additional files

Additional file 1: Table S1. CTCF ChIP-seq data. Cell lines and statistics for the ChIP-seq data used in the study. (DOCX 55 kb) Additional file 2:
**Supplementary information.** (DOCX 19 kb) Additional file 3: Figure S1. Statistics of the CTCF motif variations discovery procedure. (A) The count of sequences in Seqm at each. (B) The similarity between two continuous sequence pools Seqm-1 and Seqm. (TIFF 1426 kb) Additional file 4: Dataset S1. The PWM matrices for the three motifs. (ZIP 1.04 KB) Additional file 5: Figure S2. The binding affinities among CTCF-A, CTCF-B, CTCF-C differ significantly. (PDF 33 kb) Additional file 6: Table S2. Histone modification ChIP-seq data. Filenames and URLs for the histone modification ChIP-seq data used in the study. (DOCX 13 kb) Additional file 7: Figure S3. The distribution of different histone marks on three CTCF variations in GM12878. CTCF-A bindings are more associated with active histione modifications. (“****”, “***”, “**” and “*” represents *P*-value < 1e-5, < 1e-4, <0.001, and < 0.05, respectively. “Con” and “TS” denotes constitutive and tissue-specific CTCF bindings sites, respectively). (TIFF 1469 kb) Additional file 8: Figure S4. Distribution of the three CTCF motif variations in promoter, intergenic and intragenic regions. (TIFF 1494 kb) Additional file 9: Table S3. Transcription factor binding site ChIP-seq Data. Filenames and URLs for the transcription factor binding site ChIP-seq data used in the study. (DOCX 11 kb) Additional file 10: Figure S5. CpG coverage (%) distribution within regions [-50 bp, +50 bp] of the center of CTCF-A, CTCF-B and CTCF-C binding sites in three cell lines (GM12878, K562 and HeLaS3). (TIFF 1674 kb) Additional file 11: Figure S6. DNA methylation distribution within regions [-50 bp, +50 bp] of the center of CTCF-A, CTCF-B and CTCF-C binding sites in three cell lines (GM12878, K562 and HeLaS3). (TIFF 1717 kb) Additional file 12: Figure S7. Comparison of DNA methylation levels in bound and unbound CTCF-A motifs (Control set). The control set was extracted from CTCF-A motifs with the lowest (the bottom 5 %) reads coverage from the ChIP-seq experiments (“****” represents *P*-value < 1e-5). (TIFF 3729 kb) Additional file 13: Figure S8. Enrichment of CTCF binding at the motifs with the 12th unmethylated site. The enrichment can be seen in all three classes. (PDF 1360 kb) Additional file 14: Figure S9. Correlation between DNA looping and 36 genome features in three cell lines (GM12878, K562 and HeLaS3). The panels show the distribution of Pearson’s correlations coefficient for each genomic feature. (TIFF 8.44 MB) Additional file 15: Figure S10. Enrichment of regulatory elements in 5C fragments. CTCF-A occupied 5C distal fragments enriched with active enhancer elements (P300, H3K4me1 and annotated enhancers, * represents hypergeometric enrichment *p*-value < 0.01). (TIFF 8647 kb)

## Abbreviations

CTCF: The CCCTC-binding factor
ChIP-seq: Chromatin immunoprecipitation followed by high-throughput sequencing
TSS: Transcriptional start sites
AL: CTCF-A-Linked
BL: CTCF-B-Linked
CL: CTCF-C-Linked
LAD: Lamina associated domains
5C: Chromosome Conformation Capture Carbon Copy
C/EBP: CCAAT/Enhancer Binding Protein
