# Supplementary material for: Functional diversity of CTCFs is encoded in their binding motifs
Source: BMC Genomics. 2015 Aug 28;16(1):649. doi: 10.1186/s12864-015-1824-6 (PMC4552278; doi:10.1186/s12864-015-1824-6)
Supplement: Additional file 1: Table S1. — CTCF ChIP-seq data. Cell lines and statistics for the ChIP-seq data used in the study. (DOCX 55 kb) [file 12864_2015_1824_MOESM1_ESM.docx]

| **Cell Lines** | **Source** | **Control** | **# of replicate** | **Treatment** |
| --- | --- | --- | --- | --- |
| A549 | UW | Standard | 2 | Non |
| Bj | UW | Standard | 2 | Non |
| Caco2 | UW | Standard | 2 | Non |
| GM06690 | UW | Standard | 2 | Non |
| GM12864 | UW | Standard | 2 | Non |
| GM12878 | UW | Standard | 2 | Non |
| HBMEC | UW | Standard | 2 | Non |
| HeepiC | UW | Standard | 2 | Non |
| HelaS3 | UW | Standard | 2 | Non |
| HRE | UW | Standard | 2 | Non |
| K562 | UW | Standard | 2 | Non |
| SAEC | UW | Standard | 2 | Non |
| Input | UW | Standard | 1 | Non |
| Gm12878 | SYDH | Standard | 2 | Non |
| K562 | SYDH | Standard | 2 | Non |
| Input | SYDH | Standard | 1 | Non |
| UW:http://hgdownload.cse.ucsc.edu/goldenPath/hg19/encodeDCC/wgEncodeUwTfbs/  SYDH:http://hgdownload.cse.ucsc.edu/goldenPath/hg19/encodeDCC/wgEncodeSydhTfbs/ | | | | |
