# Supplementary material for: Functional diversity of CTCFs is encoded in their binding motifs
Source: BMC Genomics. 2015 Aug 28;16(1):649. doi: 10.1186/s12864-015-1824-6 (PMC4552278; doi:10.1186/s12864-015-1824-6)
Supplement: Additional file 2: — Supplementary information. (DOCX 19 kb) [file 12864_2015_1824_MOESM2_ESM.docx]

**Suplementary information**

**Title: Functional diversity of CTCFs is encoded in their binding motifs**

Authors: Rongxin Fang^1,#^, Chengqi Wang^1,#^, Geir Skogerbo^2^ , Zhihua Zhang^1*^

Affiliations:

1 CAS Key Laboratory of Genome Sciences and Information, Beijing Institute of Genomics, Chinese Academy of Sciences, Beijing 100101, China.

2. Bioinformatics Laboratory and National Laboratory of Biomacromolecules, Institute of Biophysics, Chinese Academy of Sciences, Beijing, China,

#. Those authors contributed equally to this work.

* To whom correspondence should be addressed.Tel: +86-10-84097249; Fax: +86-10-84097720; Email: [zhangzhihua@big.ac.cn](mailto:zhangzhihua@big.ac.cn) to ZZ

Running title:

Functional diversity of CTCF and binding motifs

Key words: CTCF; binding motif; DNA methylation; Chromatin interaction.

**The workflow of motif detection.**

The work flow of motif detection is described in pseudocode (Table S2). Some detailed information can be found below.

*Motif Generation*

Motif generation was accomplished by using the MEME-ChIP software(Machanick and Bailey 2011) available online (http://meme.nbcr.net/meme/), which was designed especially for discovering motifs in large sets of short DNA sequences centered on locations of interest. At each run, every sequence was considered to have zero or one target motif on either strand. Every run produced a single motif with the highest confidence as the output motif.

*Motif Evaluation*

The reliability of the generated motif was evaluated from two aspects. First, it should possess high confidence, i.e, the E-value reported by MEME-ChIP, which is the estimate of the number of motifs one would expect to find by chance if the letters in the input sequences are shuffled, should be small enough (less than 0.001 here). Motifs with such small E-value (<0.001) were very unlikely to be a random sequence artifact (1). Second, the detected

motif should have enough similarity to the target motif, as previously reported in the public motif databases, to prevent the production of a cofactor’s motif. Motif was compared against JASPAR (2,3) and UniPROBE databases (4) by TOMTOM (5). Motif was seen as sufficiently similar to the target if its minimum False Discovery Rate (FDR) (TOMTOM q-value) returned by TOMTOM was less than 0.01. If the above two conditions were satisfied simultaneously, the discovered motif was reported as reliable.

*Sequence Elimination*

Given the motif produced by Motif Generation, the sequence scanning tool FIMO (6) was utilized to find motif occurrence on both strands of each sequence. Motif occurrence was seen as confident if the q-value returned by FIMO was less than 0.01. Those sequences containing no occurrence and FIMO q-value<0.01 were discarded contemporarily.

*Stopping Criteria*

Motif discovery iteration was considered broken if either one of following two conditions was met: (1) similarity between two consecutive sequence sets Seq_i_ and Seq_i+1_ was greater than 98%. The similarity was defined as shown below:

$$similarity(seq_{i},seq_{i+1})=(seq_{i}\cap seq_{i+1})/(seq_{i}\cup seq_{i+1})$$

(2) the size of Seq_i_ was less than 10% of initial sequence set Seq_0_. If (2) was met at any time, the whole workflow was terminated.

1. Machanick, P. and Bailey, T.L. (2011) MEME-ChIP: motif analysis of large DNA datasets. *Bioinformatics*, **27**, 1696-1697.

2. Sandelin, A., Alkema, W., Engstrom, P., Wasserman, W.W. and Lenhard, B. (2004) JASPAR: an open-access database for eukaryotic transcription factor binding profiles. *Nucleic Acids Res*, **32**, D91-94.

3. Mathelier, A., Zhao, X., Zhang, A.W., Parcy, F., Worsley-Hunt, R., Arenillas, D.J., Buchman, S., Chen, C.Y., Chou, A., Ienasescu, H. *et al.* (2014) JASPAR 2014: an extensively expanded and updated open-access database of transcription factor binding profiles. *Nucleic Acids Res*, **42**, D142-147.

4. Robasky, K. and Bulyk, M.L. (2011) UniPROBE, update 2011: expanded content and search tools in the online database of protein-binding microarray data on protein-DNA interactions. *Nucleic Acids Res*, **39**, D124-128.

5. Gupta, S., Stamatoyannopoulos, J., Bailey, T. and Noble, W. (2007) Quantifying similarity between motifs. *Genome Biol*, **8**, R24.

6. Grant, C.E., Bailey, T.L. and Noble, W.S. (2011) FIMO: scanning for occurrences of a given motif. *Bioinformatics*, **27**, 1017-1018.
