# Supplementary figures and images for: Functional diversity of CTCFs is encoded in their binding motifs
Source: BMC Genomics. 2015 Aug 28;16(1):649. doi: 10.1186/s12864-015-1824-6 (PMC4552278; doi:10.1186/s12864-015-1824-6)

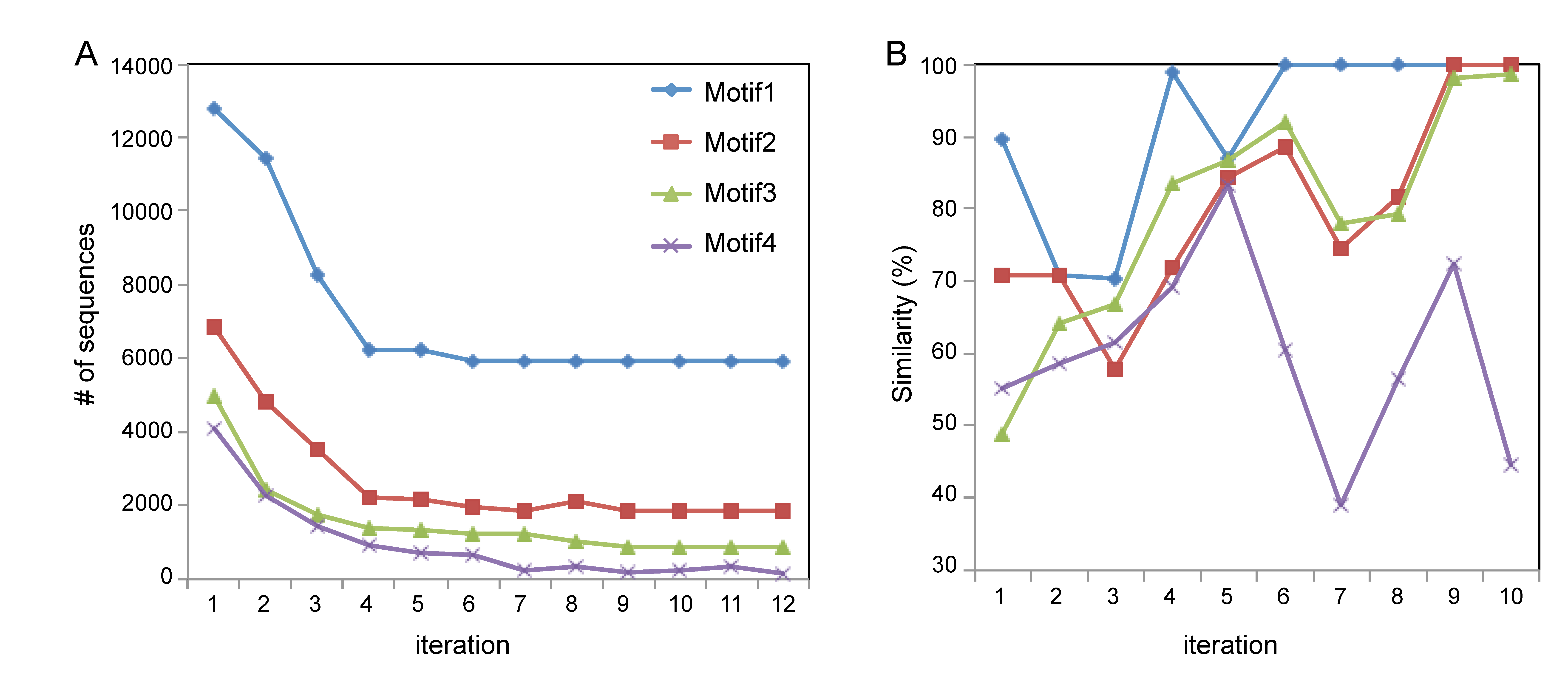

Supplement: Additional file 3: Figure S1. — Statistics of the CTCF motif variations discovery procedure. (A) The count of sequences in Seqm at each. (B) The similarity between two continuous sequence pools Seqm-1 and Seqm. (TIFF 1426 kb) [file 12864_2015_1824_MOESM3_ESM.tiff]

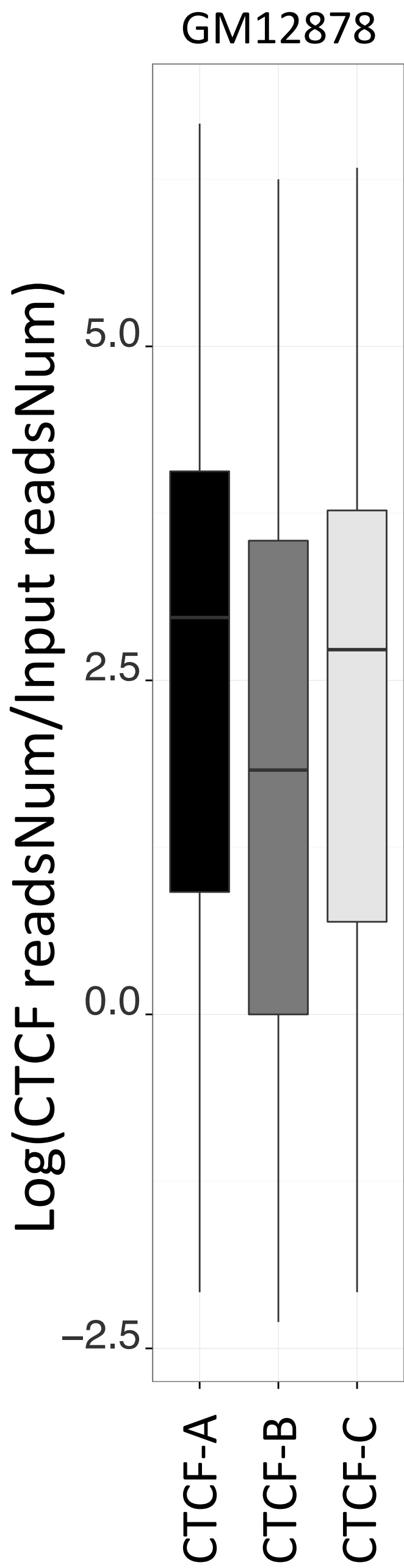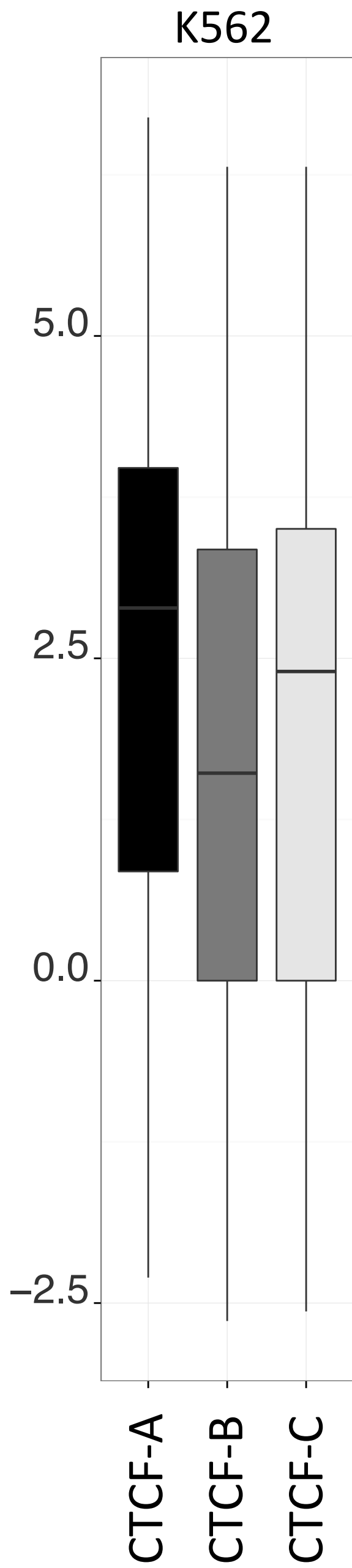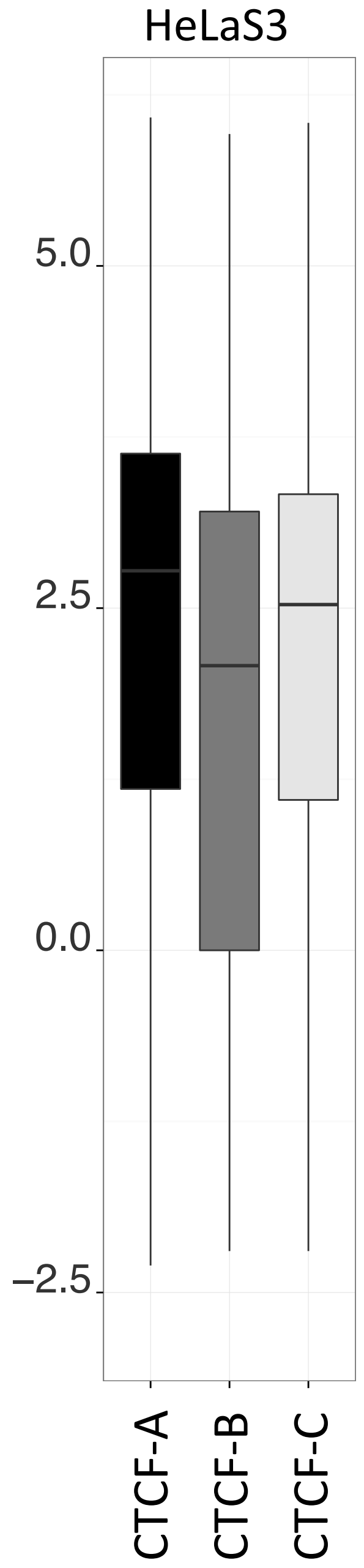

Supplement: Additional file 5: Figure S2. — The binding affinities among CTCF-A, CTCF-B, CTCF-C differ significantly. (PDF 33 kb) [file 12864_2015_1824_MOESM5_ESM.pdf]

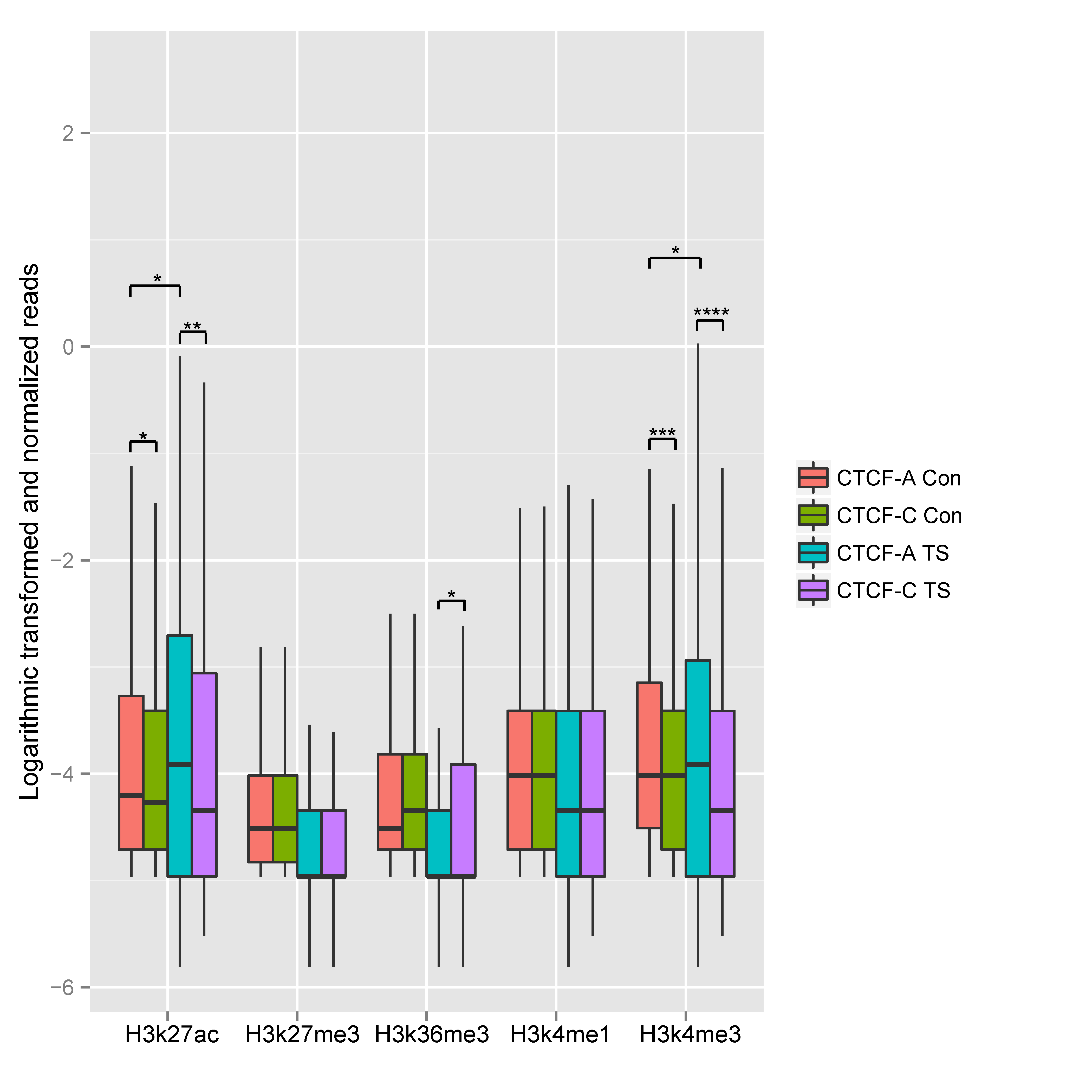

Supplement: Additional file 7: Figure S3. — The distribution of different histone marks on three CTCF variations in GM12878. CTCF-A bindings are more associated with active histione modifications. (“****”, “***”, “**” and “*” represents P-value < 1e-5, < 1e-4, <0.001, and < 0.05, respectively. “Con” and “TS” denotes constitutive and tissue-specific CTCF bindings sites, respectively). (TIFF 1469 kb) [file 12864_2015_1824_MOESM7_ESM.tiff]

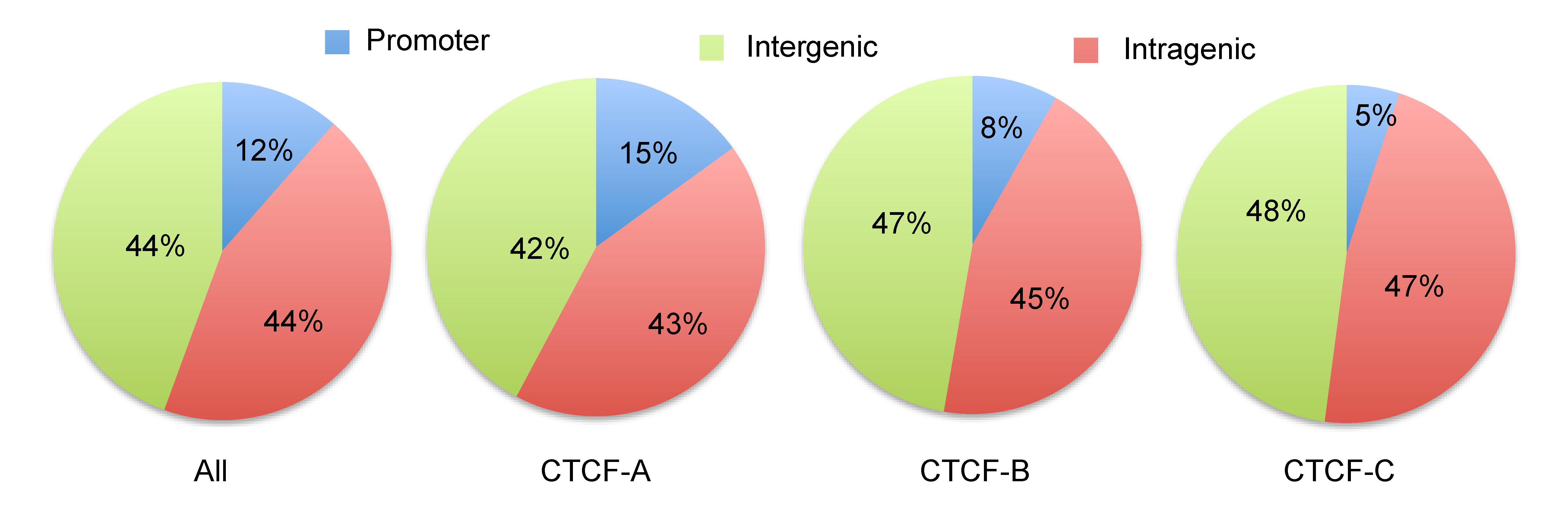

Supplement: Additional file 8: Figure S4. — Distribution of the three CTCF motif variations in promoter, intergenic and intragenic regions. (TIFF 1494 kb) [file 12864_2015_1824_MOESM8_ESM.tiff]

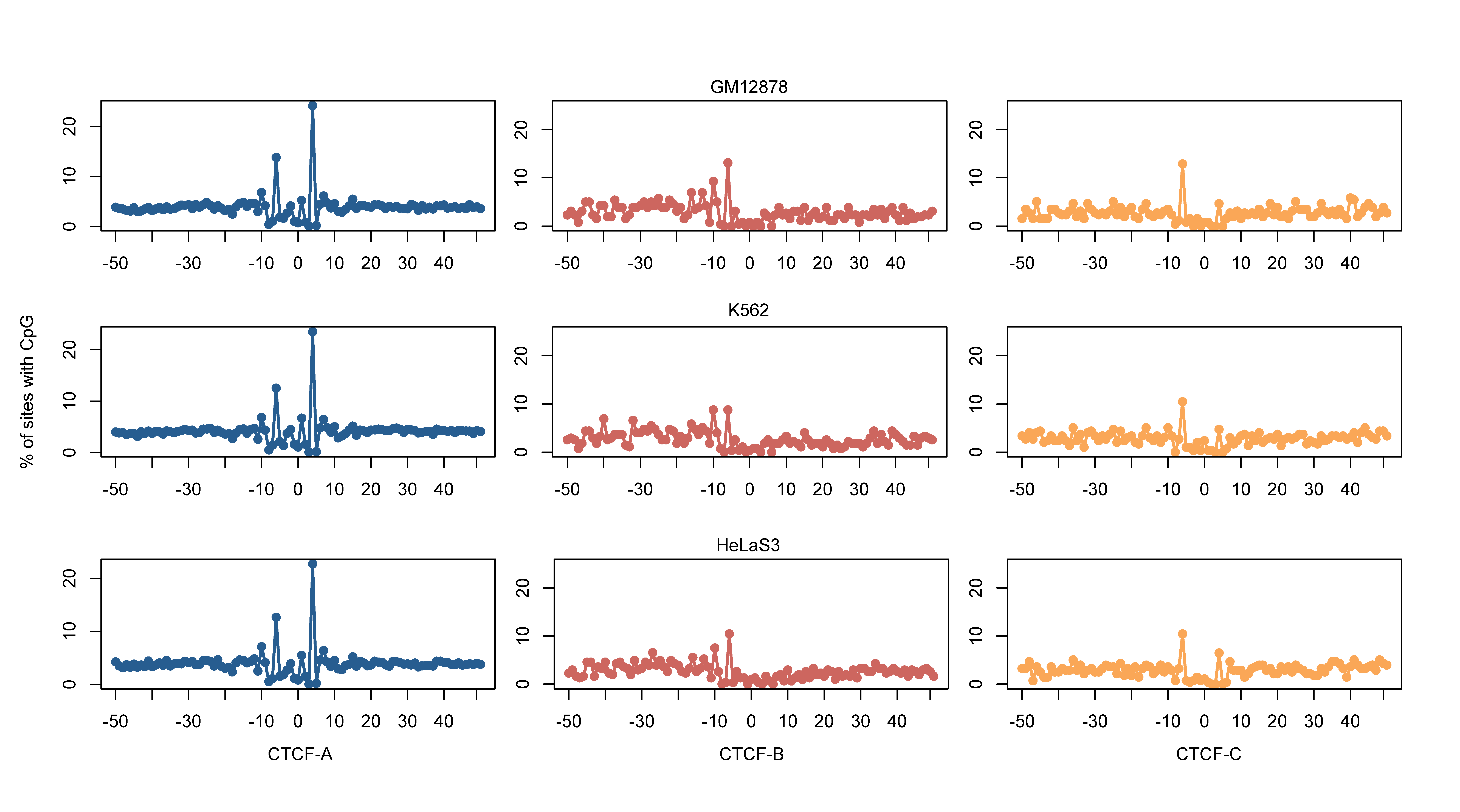

Supplement: Additional file 10: Figure S5. — CpG coverage (%) distribution within regions [-50 bp, +50 bp] of the center of CTCF-A, CTCF-B and CTCF-C binding sites in three cell lines (GM12878, K562 and HeLaS3). (TIFF 1674 kb) [file 12864_2015_1824_MOESM10_ESM.tiff]

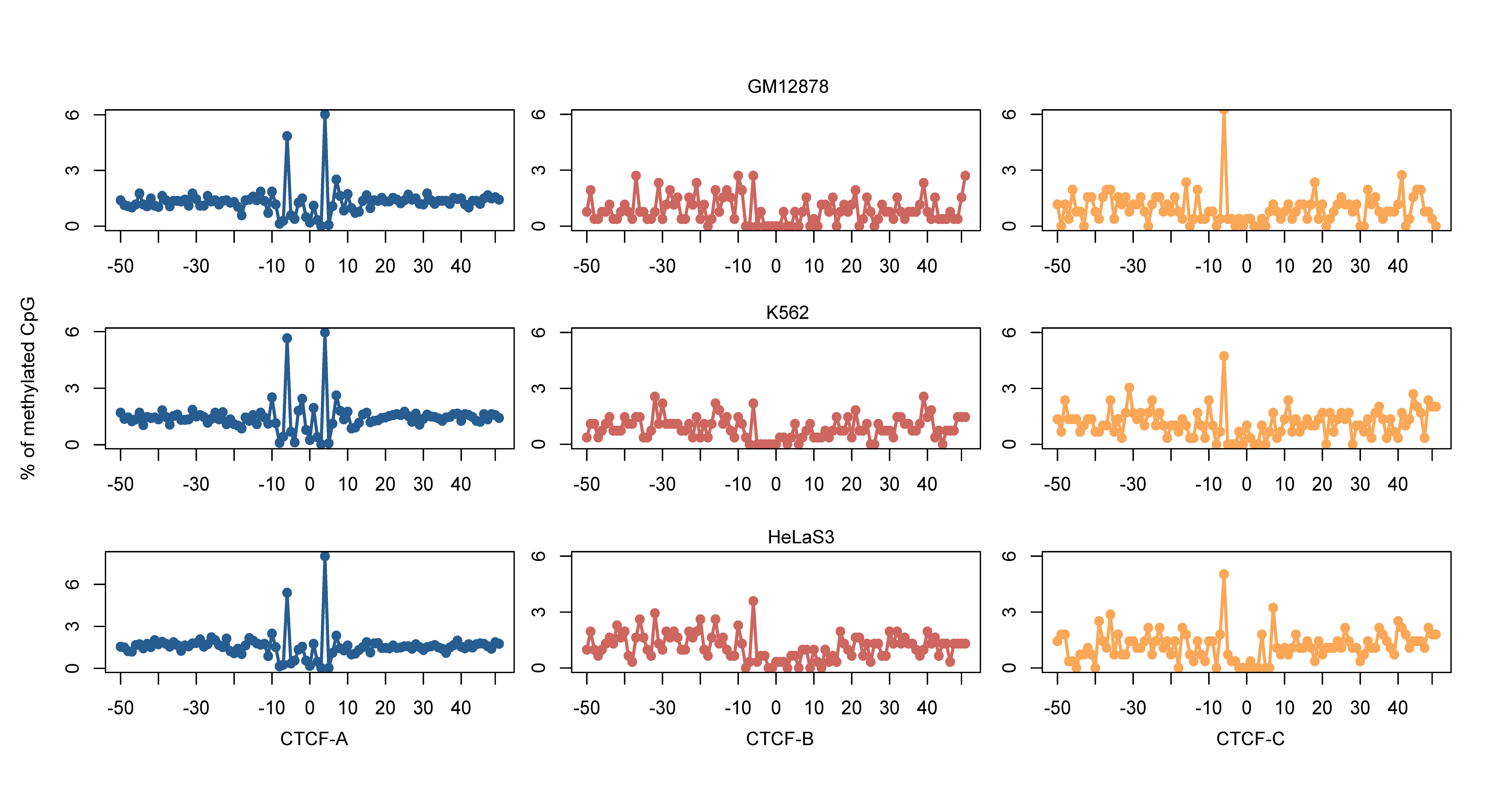

Supplement: Additional file 11: Figure S6. — DNA methylation distribution within regions [-50 bp, +50 bp] of the center of CTCF-A, CTCF-B and CTCF-C binding sites in three cell lines (GM12878, K562 and HeLaS3). (TIFF 1717 kb) [file 12864_2015_1824_MOESM11_ESM.tiff]

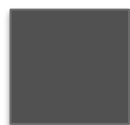

12th methylated CTCF motif

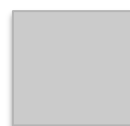

12th unmethylated CTCF motif

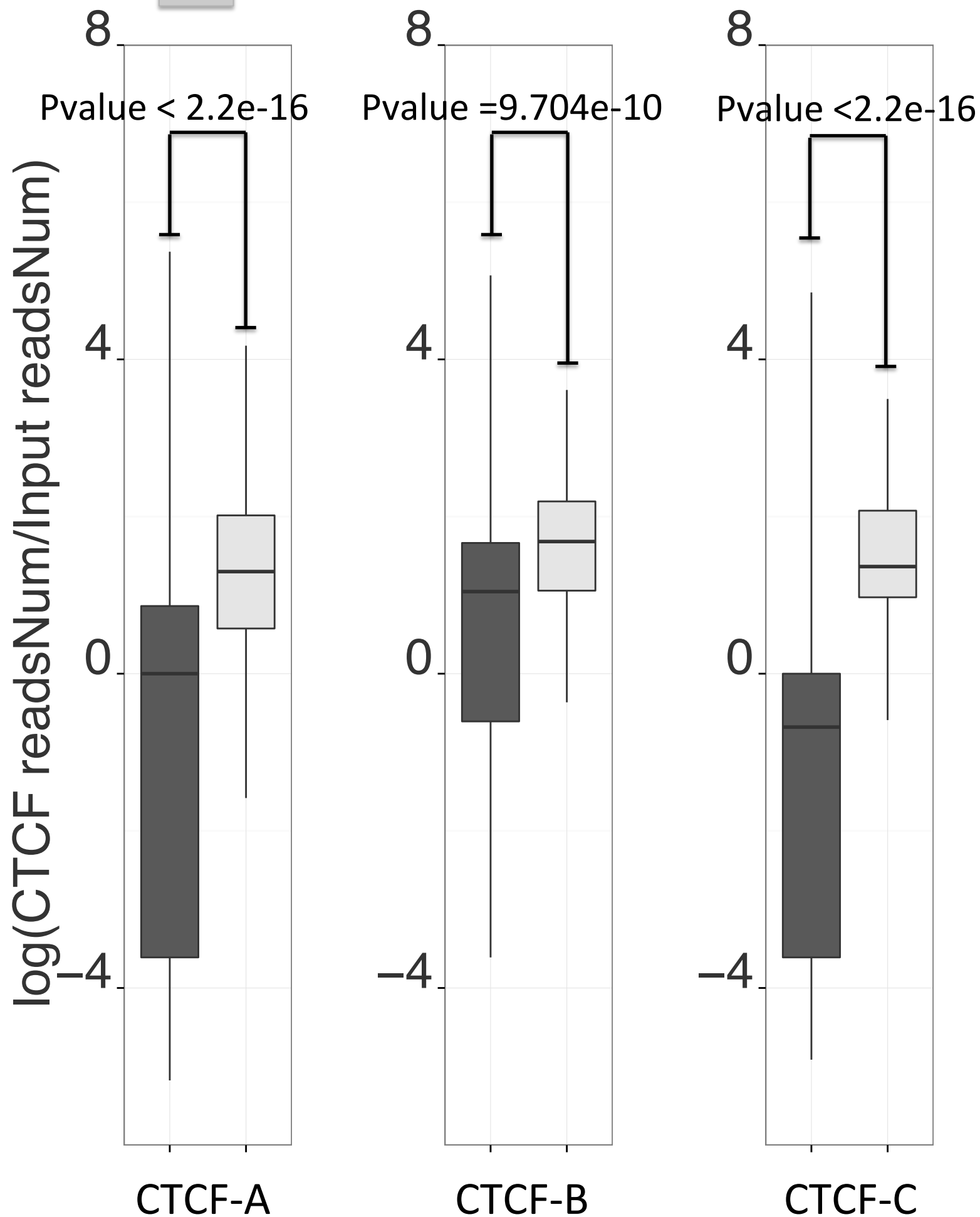

Supplement: Additional file 13: Figure S8. — Enrichment of CTCF binding at the motifs with the 12th unmethylated site. The enrichment can be seen in all three classes. (PDF 1360 kb) [file 12864_2015_1824_MOESM13_ESM.pdf]

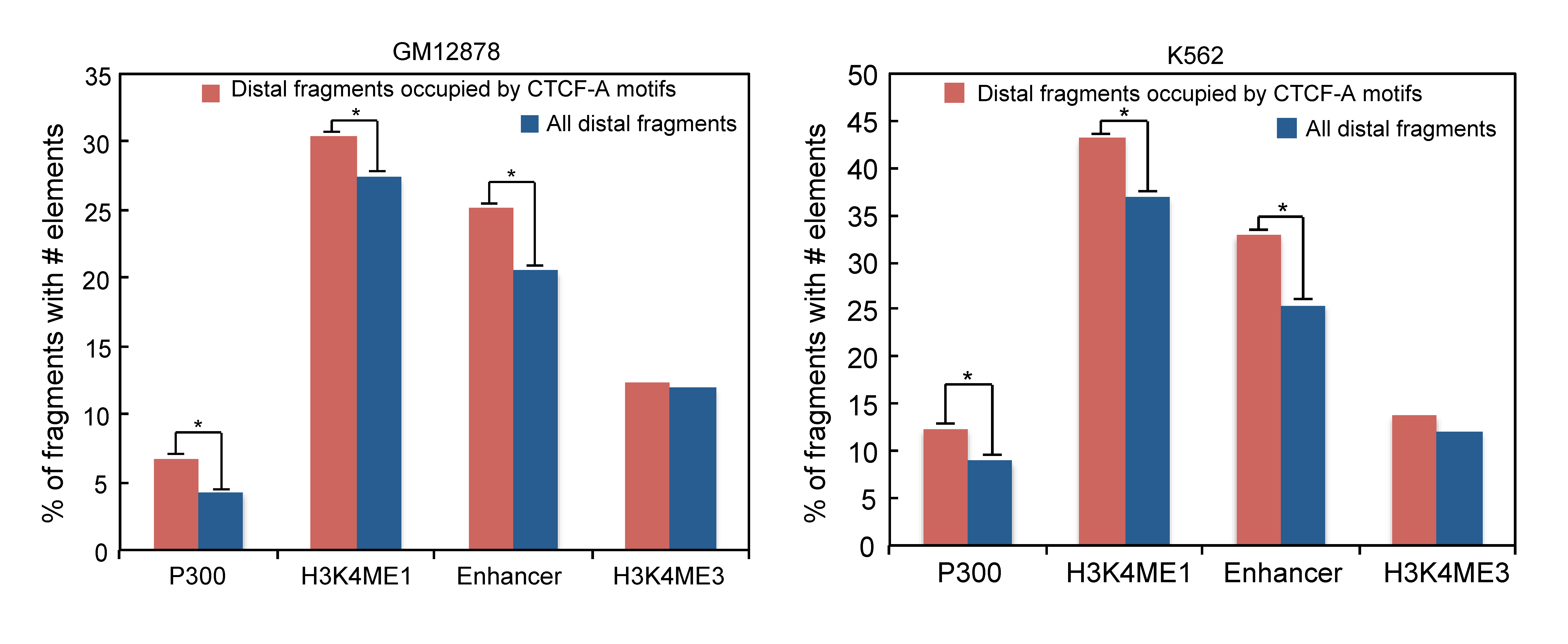

Supplement: Additional file 15: Figure S10. — Enrichment of regulatory elements in 5C fragments. CTCF-A occupied 5C distal fragments enriched with active enhancer elements (P300, H3K4me1 and annotated enhancers, * represents hypergeometric enrichment p-value < 0.01). (TIFF 8647 kb) [file 12864_2015_1824_MOESM15_ESM.tiff]
