# Supplementary material for: Functional diversity of CTCFs is encoded in their binding motifs
Source: BMC Genomics. 2015 Aug 28;16(1):649. doi: 10.1186/s12864-015-1824-6 (PMC4552278; doi:10.1186/s12864-015-1824-6)
Supplement: Additional file 6: Table S2. — Histone modification ChIP-seq data. Filenames and URLs for the histone modification ChIP-seq data used in the study. (DOCX 13 kb) [file 12864_2015_1824_MOESM6_ESM.docx]

|  | |
| --- | --- |
| **Name** | **File name** |
| Control | wgEncodeBroadHistoneGm12878ControlStdSig.bigWig |
| H2AZ | wgEncodeBroadHistoneGm12878H2azStdSig.bigWig |
| H3K4me1 | wgEncodeBroadHistoneGm12878H3k4me1StdSig.bigWig |
| H3K4me2 | wgEncodeBroadHistoneGm12878H3k4me2StdSig.bigWig |
| H3K4me3 | wgEncodeBroadHistoneGm12878H3k4me3StdSig.bigWig |
| H3K9ac | wgEncodeBroadHistoneGm12878H3k9acStdSig.bigWig |
| H3K9me3 | wgEncodeBroadHistoneGm12878H3k9me3StdSig.bigWig |
| H3K27ac | wgEncodeBroadHistoneGm12878H3k27acStdSig.bigWig |
| H3K27me3 | wgEncodeBroadHistoneGm12878H3k27me3StdSig.bigWig |
| H3K36me3 | wgEncodeBroadHistoneGm12878H3k36me3StdSig.bigWig |
| H3K79me2 | wgEncodeBroadHistoneGm12878H3k79me2StdSig.bigWig |
| H3K20me1 | wgEncodeBroadHistoneGm12878H4k20me1StdSig.bigWig |
| URL:http://hgdownload.cse.ucsc.edu/goldenPath/hg19/encodeDCC/wgEncodeBroadHistone/ | |
