# Supplementary material for: Functional diversity of CTCFs is encoded in their binding motifs
Source: BMC Genomics. 2015 Aug 28;16(1):649. doi: 10.1186/s12864-015-1824-6 (PMC4552278; doi:10.1186/s12864-015-1824-6)
Supplement: Additional file 9: Table S3. — Transcription factor binding site ChIP-seq Data. Filenames and URLs for the transcription factor binding site ChIP-seq data used in the study. (DOCX 11 kb) [file 12864_2015_1824_MOESM9_ESM.docx]

|  | | |
| --- | --- | --- |
| **Name** | **File name** | **Source** |
| POL2 | wgEncodeOpenChromChipGm12878Pol2Pk.narrowPeak.gz | UTA |
| C-MYC | wgEncodeOpenChromChipGm12878CmycPk.narrowPeak.gz | UTA |
| CHD2 | wgEncodeSydhTfbsGm12878Chd2ab68301IggmusPk.narrowPeak.gz | SYDH |
| COREST | wgEncodeSydhTfbsGm12878Corestsc30189IggmusPk.narrowPeak.gz | SYDH |
| E2F4 | wgEncodeSydhTfbsGm12878E2f4IggmusPk.narrowPeak.gz | SYDH |
| ELK1 | wgEncodeSydhTfbsGm12878Elk112771IggmusPk.narrowPeak.gz | SYDH |
| MAFK | wgEncodeSydhTfbsGm12878MafkIggmusPk.narrowPeak.gz | SYDH |
| MXI1 | wgEncodeSydhTfbsGm12878Mxi1IggmusPk.narrowPeak.gz | SYDH |
| NFYA | wgEncodeSydhTfbsGm12878NfyaIggmusPk.narrowPeak.gz | SYDH |
| NFYB | wgEncodeSydhTfbsGm12878NfybIggmusPk.narrowPeak.gz | SYDH |
| NRF1 | wgEncodeSydhTfbsGm12878Nrf1IggmusPk.narrowPeak.gz | SYDH |
| RFX5 | wgEncodeSydhTfbsGm12878Rfx5200401194IggmusPk.narrowPeak.gz | SYDH |
| SMC3 | wgEncodeSydhTfbsGm12878Smc3ab9263IggmusPk.narrowPeak.gz | SYDH |
| STAT1 | wgEncodeSydhTfbsGm12878Stat1StdPk.narrowPeak.gz | SYDH |
| TBP | wgEncodeSydhTfbsGm12878TbpIggmusPk.narrowPeak.gz | SYDH |
| USF2 | wgEncodeSydhTfbsGm12878Usf2IggmusPk.narrowPeak.gz | SYDH |
| ZNF143 | wgEncodeSydhTfbsGm12878Znf143166181apStdPk.narrowPeak.gz | SYDH |
| ZNF274 | wgEncodeSydhTfbsGm12878Znf274StdPk.narrowPeak.gz | SYDH |
| P300 | wgEncodeSydhTfbsGm12878P300sc584IggmusPk.narrowPeak.gz | SYDH |
| CFOS | wgEncodeSydhTfbsGm12878CfosStdPk.narrowPeak.gz | SYDH |
| JUND | wgEncodeSydhTfbsGm12878JundStdPk.narrowPeak.gz | SYDH |
| MAX | wgEncodeSydhTfbsGm12878MaxStdPk.narrowPeak.gz | SYDH |
| RAD21 | wgEncodeSydhTfbsGm12878Rad21IggrabPk.narrowPeak.gz | SYDH |
| TR4 | wgEncodeSydhTfbsGm12878Tr4StdPk.narrowPeak.gz | SYDH |
| UTA: http://hgdownload.cse.ucsc.edu/goldenPath/hg19/encodeDCC/wgEncodeOpenChromChip/  SYDH: http://hgdownload.cse.ucsc.edu/goldenPath/hg19/encodeDCC/wgEncodeSydhTfbs/ | | |
